# The impact of STAT3 and phospho-STAT3 expression on the prognosis and clinicopathology of ovarian cancer: a systematic review and meta-analysis

**DOI:** 10.1186/s13048-021-00918-6

**Published:** 2021-11-18

**Authors:** Shuo Gao, Wenyuan Zhang, Na Yan, Min Li, Xiaowei Mu, Huaxia Yin, Jinhua Wang

**Affiliations:** 1 Graduate School of Inner Mongolia Medical University, Hohhot, 010059 Inner Mongolia Autonomous Region China; 2 ECG Network Center of Special Inspection Department, Dezhou Municipal Hospital, Dezhou, 253000 Shandong China; 3 Department of Pathology, Tumor Hospital of Inner Mongolia Autonomous Region, Hohhot, 010010 Inner Mongolia Autonomous Region China

**Keywords:** Ovarian cancer, Meta-analysis, STAT3/p-STAT3, Prognosis

## Abstract

**Purpose:**

STAT3 and p-STAT3 are often overexpressed in various human tumours and participate in cancer development and progression. However, whether STAT3/p-STAT3 expression is associated with clinicopathologic characteristics and has prognostic significance for people suffering from ovarian cancer remains controversial. We conducted a systematic review and meta-analyses to clarify the associations between STAT3/p-STAT3 expression and clinicopathologic characteristics and prognostic factors of ovarian cancer.

**Methods:**

A systematic electronic search in the PubMed, Embase, CNKI, and Wanfang databases was conducted to identify relevant articles published before 3 April 2021. All statistical analyses were performed using Stata 15.1.

**Results:**

We included 16 eligible studies incorporating 1747 ovarian cancer patients. The expression of STAT3/p-STAT3 was upregulated in ovarian cancer samples versus normal ovarian tissue, benign tumours and borderline tumours (OR = 10.14, *p* < 0.00001; OR = 9.08, *P* < 0.00001; OR = 4.01, *p* < 0.00001, respectively). STAT3/p-STAT3 overexpression was significantly correlated with FIGO stage (I-II vs. III-IV) (OR = 0.36, *p* < 0.00001), tumour grade (G1 + G2 vs. G3) (OR = 0.55; *p* = 0.001) and lymph node metastasis (yes vs. no) (OR = 3.39; *p* < 0.00001). High STAT3/p-STAT3 expression was correlated with poorer prognosis of ovarian cancer patients for both overall survival (OS) (HR = 1.67, *p* < 0.00001) and progression-free survival (PFS) (HR = 1.40, *p* = 0.007).

**Conclusion:**

The present meta-analysis indicated that high STAT3/p-STAT3 expression is likely predictive of an unfavourable prognosis in ovarian cancer patients. Nonetheless, prospective trials are required to confirm these associations.

**Supplementary Information:**

The online version contains supplementary material available at 10.1186/s13048-021-00918-6.

## Introduction

Ovarian cancer constitutes the most lethal gynaecological tumour type, accounting for approximately 125,000 deaths annually worldwide [1, 2]. Despite tremendous efforts in improving treatment modalities, such as platinum-based anticancer therapy, new biological therapies, and surgical techniques, the 5-year mortality rate of advanced ovarian cancer is approximately 30–40% due to the lack of new diagnosis and screening practices [3]. Currently, prognostic factors used in clinical practice remain pathological variables, such as the International Federation of Gynecology and Obstetrics (FIGO) stage, tumour grade, tumour-node-metastasis, histologic subtype, and overall survival (OS). Studies have focused on researching various molecular signal responses or pathways in ovarian cancer for early screening and prognostic evaluation. Thus, the development of a more reliable biomarker for disease progression is urgently required.

The signal transducers and activators of transcription (STAT) family comprises seven members encoded by different genes: STAT1, STAT2, STAT3, STAT4, STAT5a, STAT5b, and STAT6, among which STAT3 is closely related to carcinogenesis [4, 5]. STAT3 signal transduction begins with the binding of extracellular ligands to cell surface receptors, leading to receptor dimerization and transphosphorylation of Janus kinase (JAK) tyrosine residues and JAK activation [6]. The tail end of the cytoplasmic receptor provides a binding site for STAT3. Subsequently, JAK activates tyrosine 705 at the C-terminus of STAT3. Activated STAT3 is isolated from the receptor/kinase complex and interacts with SH2 to form STAT3: STAT3 homodimers or STAT3: STAT1 heterodimers, which have a total of 9 bp of DNA [7]. The specific response elements in the sequence (TTCNNNGAA) interact to induce transcription of target genes vital to physiological and pathological functions, regulating cellular development, differentiation, proliferation, apoptosis, invasion, and metastasis [8, 9]. Under physiological conditions, the activation of the STAT3 signalling pathway is short-lived and can quickly return to an inactivated state to prevent the unintended regulation of genes, which causes many human diseases. The inactivation of negative regulation, the overstimulation of STAT3, and the continuous activation of the positive feedback loop can lead to the constitutive activation of STAT3. This phenomenon is usually observed in cancer patients.

Several recently published meta-analyses have suggested that the expression levels of STAT3 and p-STAT3 are promising prognostic biomarkers for glioma [10], breast [11, 12], lung [13, 14], and colorectal carcinoma [15]. Therefore, STAT3/p-STAT3 could serve as a new clinicopathological marker for a poor prognosis in many human malignancies. Some retrospective studies have been published that assessed the role of STAT3/p-STAT3 expression in ovarian cancer but have generated conflicting results. To address this contradiction, we systematically searched for available literature and conducted a meta-analysis to assess the clinicopathological value and prognostic ability of STAT3/p-STAT3 in ovarian carcinoma.

## Materials and methods

### Search strategy

A comprehensive literature search of PubMed, Embase, CNKI, and Wanfang was performed. Scientific papers reporting associations between STAT3/p-STAT3 expression and pathological characteristics of ovarian cancer and survival outcomes published prior to 3 April 2021 in English or Chinese in peer-reviewed journals were selected. We used the following keywords to search for publications (ovarian OR ovary): AND (cancer OR carcinoma OR tumour OR neoplasm) AND (STAT3 OR STAT3 transcription factor OR signal transducer and activator of transcription 3 OR STAT3 protein OR pSTAT3 OR phospho-STAT3 OR phosphorylated signal transducer and activator of transcription 3 OR phosphorylated STAT3 transcription factor OR p-STAT3 OR phosphate STAT3).

### Study selection and inclusion criteria

Studies meeting the following inclusion criteria were included in our systematic review: (1) participants were diagnosed with ovarian cancer through pathological or clinical diagnosis methods; (2) determination of STAT3 and p-STAT3 in neoplastic tissue was performed using immunohistochemical (IHC) staining; (3) the study provided data on the association between STAT3/p-STAT3 expression and survival outcomes, including hazard ratios (HRs), 95% confidence intervals (CIs), or Kaplan–Meier survival curves; (4) the study provided data on the relevance of STAT3/p-STAT3 expression to clinicopathological features, including histological type, pathological type, FIGO stage, tumour grade, and lymph node metastasis; (5) when the results of a study were published in two or more journals, we selected the most complete or the latest version; and (6) all studies were restricted to English or Chinese language publications.

The exclusion criteria were as follows: (1) reviews, expert opinions, abstracts, case reports, conference reports, systematic reviews, meta-analyses, letters, ongoing studies; and (2) studies related to cell lines, tissue culture, or animal models.

### Data extraction quality assessment

Two independent researchers (GS and ZWY) screened all titles and abstracts, and if necessary, screened the full text to determine relevant studies. Any difference in opinion was resolved through discussion by the two reviewers until consensus was reached. If the data in the text were incomplete or missing, we contacted the primary authors by email or fax to obtain the necessary data.

The extracted data comprised the name of the first author, year of publication, study country, number of patients, age, level of STAT3 or p-STAT3 expression, pathological type, histological type, FIGO stage, tumour grade, lymph node metastasis, antibody, scoring method, cut-off value, follow-up, survival analysis, and HR estimate. The quality of the included studies was evaluated using the Newcastle–Ottawa Quality Assessment Scale (NOS) [16]. The NOS has a score range of 0 to 9, with a score ≥ 7 indicating high quality.

### Statistical analyses

The relationships between STAT3/p-STAT3 expression and clinicopathologic features of ovarian cancer were assessed with odds ratios (ORs) and 95% CIs. A combination of HRs and 95% CIs was used to estimate the impact of STAT3/p-STAT3 expression in ovarian cancer on progression-free survival (PFS) and OS. When studies reported only Kaplan–Meier curves (original data were not available), we estimated HRs and their corresponding 95% CIs using Engauge Digitizer version 11.2 (https://github.com/markummitchell/engauge-digitizer/releases) and the Excel program file (http://www.biomedcentral.com/content/supplementary/1745-6215-8-16-S1.xls) provided by Tierney et al. [17]. Cochran’s Q test was used to assess heterogeneity, and an I^2^ random effects model was used to conduct the meta-analysis if the Q test *P*-value was < 0.10 or I ^2^ > 50% [18]; otherwise, a fixed effects model was used. To explore the source of heterogeneity among the studies, subgroup analysis was performed. We also performed a sensitivity analysis to assess the stability of the meta-analysis. If ≥10 studies were available, funnel plot visual inspection, the Begg rank correlation test, and Egger’s linear regression test were used to assess the potential publication bias [19]. Stata 15.1 statistical software was adopted to perform the analyses. All statistical testing was 2-sided, and *P* < 0.05 denoted statistical significance.

## Results

### Literature search and study selection

**Fig. 1:**
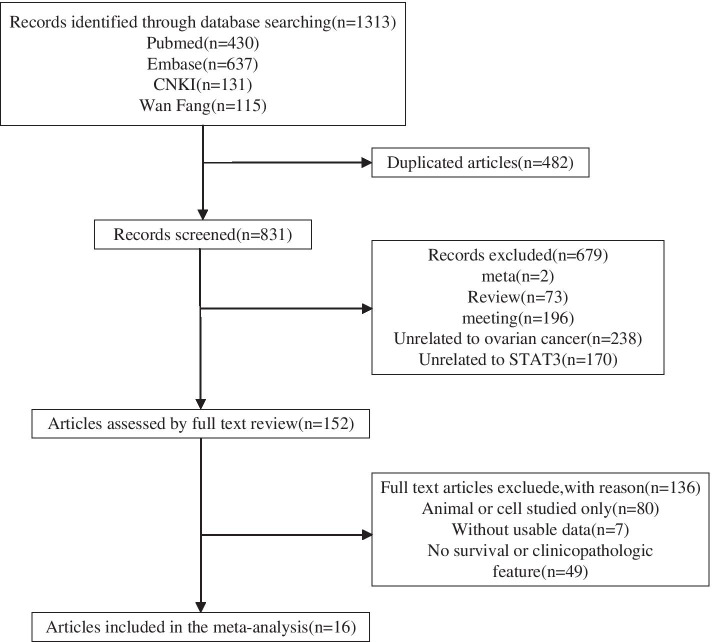
Flow chart of the study selection process

In total, 1313 articles were retrieved in the initial database search. After excluding duplicate citations and studies, we screened the titles and abstracts. The full texts were then read, and 16 publications were finally included. Figure 1 shows a detailed flow chart of the selection process.

### Study characteristics

Table 1 shows the primary features of the 16 studies included in this meta-analysis. These 16 studies included a combined total of 1747 ovarian cancer patients. Among the included studies, 13 [20, 22–30, 32, 34, 35] investigated the association between clinicopathological parameters and STAT3/p-STAT3 expression in ovarian cancer patients. Seven studies [20–24, 30, 31] assessed the association between STAT3/p-STAT3 expression and OS and PFS; 3 studies [27, 28, 33] only included patients with serous ovarian cancer, one study [31] only included patients with clear cell carcinoma. Of the 16 studies, 8 studies [20, 21, 24, 25, 29–32] were concerned with p-STAT3 expression, 6 studies [26–28, 33–35] were concerned with STAT3 expression, and 2 studies [22, 23] assessed both STAT3 and p-STAT3. Regarding location, 10 studies [22, 23, 25–30, 34, 35] were from China, 2 studies [20, 31] were from Japan, 3 studies [21, 24, 33] were from the USA and 1 study [32] was from the Netherlands. The pathological type was reported in 7 studies [22, 25–28, 34, 35] (Min’s research included both STAT3 and p-STAT3); the FIGO stage was reported in 10 studies [22–24, 26–28, 30, 32, 34, 35] (Min and Shang’s research included both STAT3 and p-STAT3); the tumour grade was reported in 7 studies [22–26, 28, 30] (Min and Shang’s research included both STAT3 and p-STAT3); lymph node metastasis was reported in 7 studies [22, 23, 25–28, 30] (Min and Shang ‘s research included both STAT3 and p-STAT3); HRs and the corresponding 95% CIs were directly extracted from the 4 original articles [20, 21, 23, 31] (Shang’s research included both STAT3 and p-STAT3), 4 studies estimated the HRs and 95% CIs using Kaplan-Meier analysis [22, 24, 30, 33] (Min’s research included both STAT3 and p-STAT3); and histological type was reported in 7 studies [20, 23, 24, 26, 29, 30, 32]. All of the included studies used IHC methods to assess STAT3/p-STAT3 expression. Regarding the scoring method, 11 studies [23–25, 27–29, 31–35] scored the expression status of STAT3/p-STAT3 based on the percentage of positive cells and the intensity of positive staining; 4 studies [21, 22, 26, 30] quantified the expression of STAT3/p-STAT3 based on the percentage of positive cells; and 1 study [20] did not mention the scoring method. A rabbit antibody was used in 6 studies [20, 23, 26, 28, 32, 34], a mouse antibody was used in 1 study [27], and the remaining 9 studies [21, 22, 24, 25, 29–31, 33, 35] did not mention the type of antibody used.

**Table 1:** Characteristics of patients included in this meta-analysis

Study	Country	N pts	Age (years)	p-STAT3+ (%)	STAT3+ (%)	Pathological type (cancer/ borderline/ benign/ normal)	Histological type (S/C/E/M)	FIGO stage (I-II/III-IV)	Tumour grade (G1 + G2/ G3)	LN (yes/no)	Anti- body	Scoring method	Cut-off value	Follow-up (months)	Survival analysis	HR estimate	NOS score
Yoshikawa (2018) [20]	Japan	341	16–82 (53 median)	95 (28%)	NR	NR	S144, M60, E52, C85	166/175	NR	NR	RmAb	NR	> 10%	median 58 (1–257)	OS/PFS	HR	8
Yang [21] (2013)	USA	49	41–87 (61 median)	25 (51.02%)	NR	NR	SME47, CU2	16/33	18/31	NR	Ab	E	≥50%	NR	OS	HR	7
Min [22] (2009)	China	50	22–73 (50.6 median)	29 (58%)	44 (88%)	50/NR/20/20	S45, M2, C2, O1	18/32	34/16	9/22	Ab	I,E^(P)^	1+, ≥10%^(P)^	NR	OS	K-M	7
Shang [23] (2017)	China	136	21–83 (54 median)	72 (52.94%)	86 (63.23%)	NR	S72, M36, E15, C13	56/80	52/84	59/77	R Ab	EI	≥4	48	OS	HR	8
Rosen [24] (2006)	USA	303	20–86 (58.2 median)	261 (86%)	NR	NR	S232, M6, E33, C15, O36	56/247	14/289	NR	Ab	EI	≥3	median 64 (1–120)	OS	K-M	8
Xiao [25] (2015)	China	40	NR	32 (80%)	NR	40/20/20/NR	NR	NR	12/28	31/9	Ab	EI	≥3	NR	NR	NR	5
Gao [26] (2013)	China	34	30–78 (55.6 median)	NR	28 (82.4%)	34/10/10/NR	S18, M10, E4, C2	12/22	19/15	20/14	RmAb	E	≥6%	NR	NR	NR	5
Wu [27] (2021)	China	130	23–67 (53 median)	NR	90 (69.2%)	130/30/20/NR	NR	47/83	NR	52/78	MAb	EI	> 1+ ≥25%	NR	NR	NR	8
Zhang [28] (2008)	China	56	34–71 (52.5 median)	NR	49 (87.5%)	56/18/35/25	NR	12/44	35/21	32/24	RpAb	EI	≥2	NR	NR	NR	6
Chen [29] (2017)	China	31	NR	10 (32.26%)	NR	NR	S15, M4, E7, C5	10/21	NR	NR	Ab	EI	≥3	NR	NR	NR	8
Li [30] (2020)	China	156	23–73 (50.6 median)	86 (55.13%)	NR	NR	S116, M22, E12, O6	46/110	64/84	43/113	Ab	E	≥50%	median 84 (60–108)	OS/PFS	K-M	7
Yanaihara [31] (2016)	Japan	84	NR	33 (39.29%)	NR	NR	NR	63/21	NR	NR	Ab	EI	> 2	NR	OS/PFS	HR	8
Wouters [32] (2015)	Netherland	101	57.99 median	20 (20%)	NR	NR	S57, M7, E18	27/74	NR	NR	RmAb	EI	≥3	NR	NR	NR	8
Chaluvally_Raghavan [33] (2016)	USA	145	35–88 (58.8 median)	NR	67 (46.2%)	NR	S145	9/136	NR/145	NR	Ab	EI	> 1	NR	OS	K-M	7
Guan [34] (2010)	China	42	37–72 (52 median)	NR	34 (80.95%)	42/NR/25/13	S29, M9, E4	9/33	NR	NR	RAb	EI	≥3	NR	NR	NR	6
Hong [35] (2017)	China	49	42–76 (63.4 median)	NR	34 (69.4%)	49/NR/14/NR	NR	35/14	NR	NR	Ab	EI	≥3	NR	NR	NR	5

*N pts* number of patients, *STAT3* signal transducer and activator of transcription 3, *p-STAT3* phospho-STAT3, *NR* not reported, *C* clear cell, *S* serous, *E* endometrioid, *M* mucinous, *U* Undifferentiated, *O* Others, *FIGO* International Federation of Gynecology and Obstetrics, *LN* Lymph node metastasis, *M* mouse, *R* rabbit, *pAb* polyclonal antibody, *mAb* monoclonal antibody, *E* extent, *I* intensity, ^*p* phospho-STAT3^, *OS* overall survival, *PFS* progression-free survival, *K-M* Kaplan–Meier survival curves, *HR* hazard ratio, *NOS* Newcastle–Ottawa Quality Assessment Scale

### Expression of STAT3/p-STAT3 in ovarian cancer versus normal ovarian tissue

**Fig. 2:**
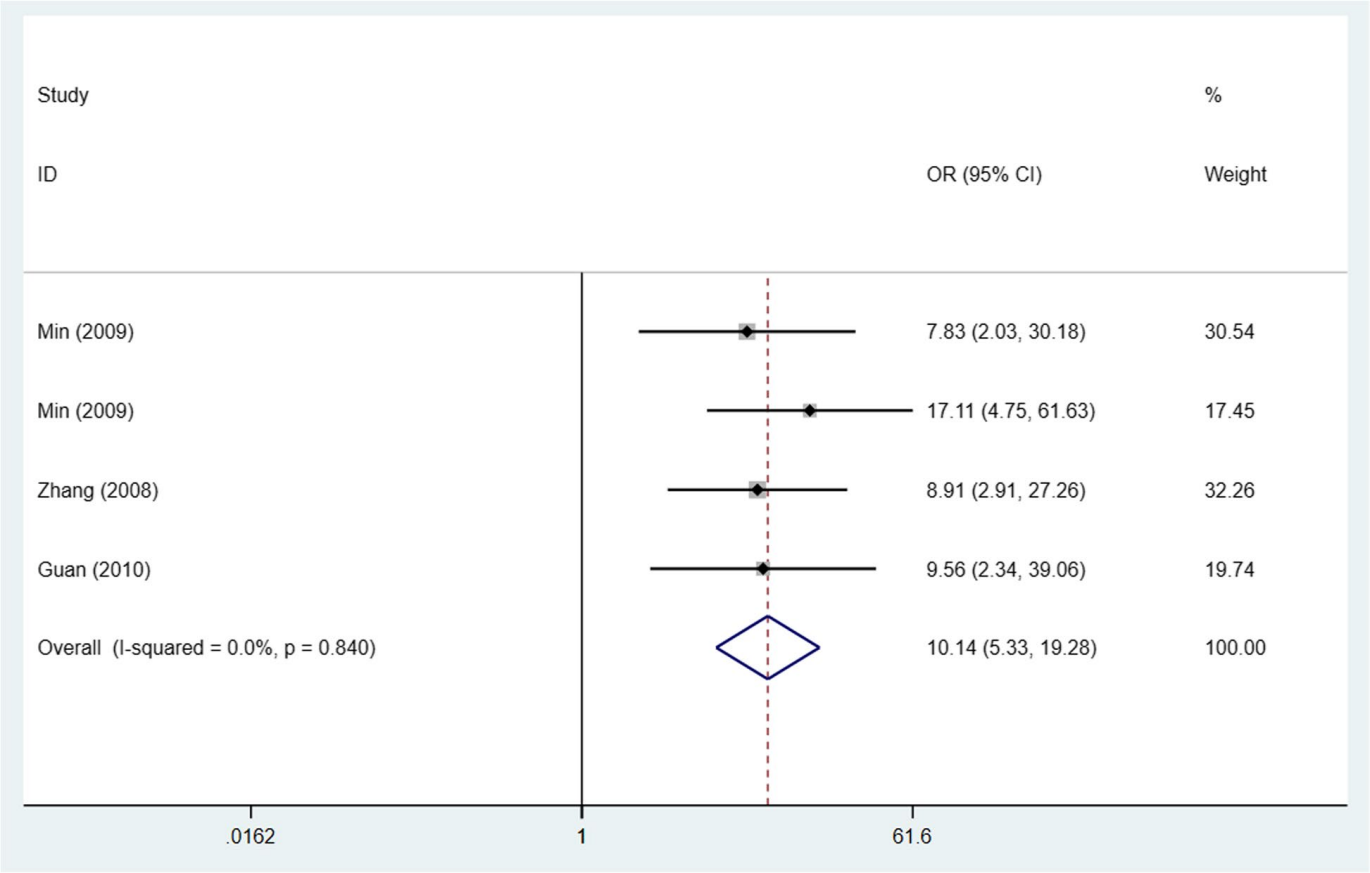
Forest plots of odds ratios for ovarian carcinoma vs. normal ovarian tissue. Abbreviation: OR, odds ratio

Data of STAT3/p-STAT3 expression in ovarian carcinoma and normal ovary tissue from 3 studies (4 trials) [22, 28, 34] were included in this meta-analysis to compare STAT3/p-STAT3 expression in ovarian carcinoma versus normal ovarian tissue. The data came from studies involving 198 ovarian cancer cases and 78 women without ovarian cancer. A fixed effects model was adopted because there was no apparent interstudy heterogeneity (I^2^ = 0%, *p* = 0.84). These studies revealed that STAT3/p-STAT3 expression was significantly higher in ovarian carcinoma tissue than in normal tissue (OR = 10.14, 95% CI = 5.33–19.28, *p* < 0.00001) (Fig. 2). A sensitivity analysis was performed to evaluate the influence of individual studies on the pooled ORs by deleting single studies in turn. According to the sensitivity analysis, the identified significant difference was robust (Additional file 1: Fig. S1).

### Expression of STAT3/p-STAT3 in ovarian cancer versus benign ovarian tumours

**Fig. 3:**
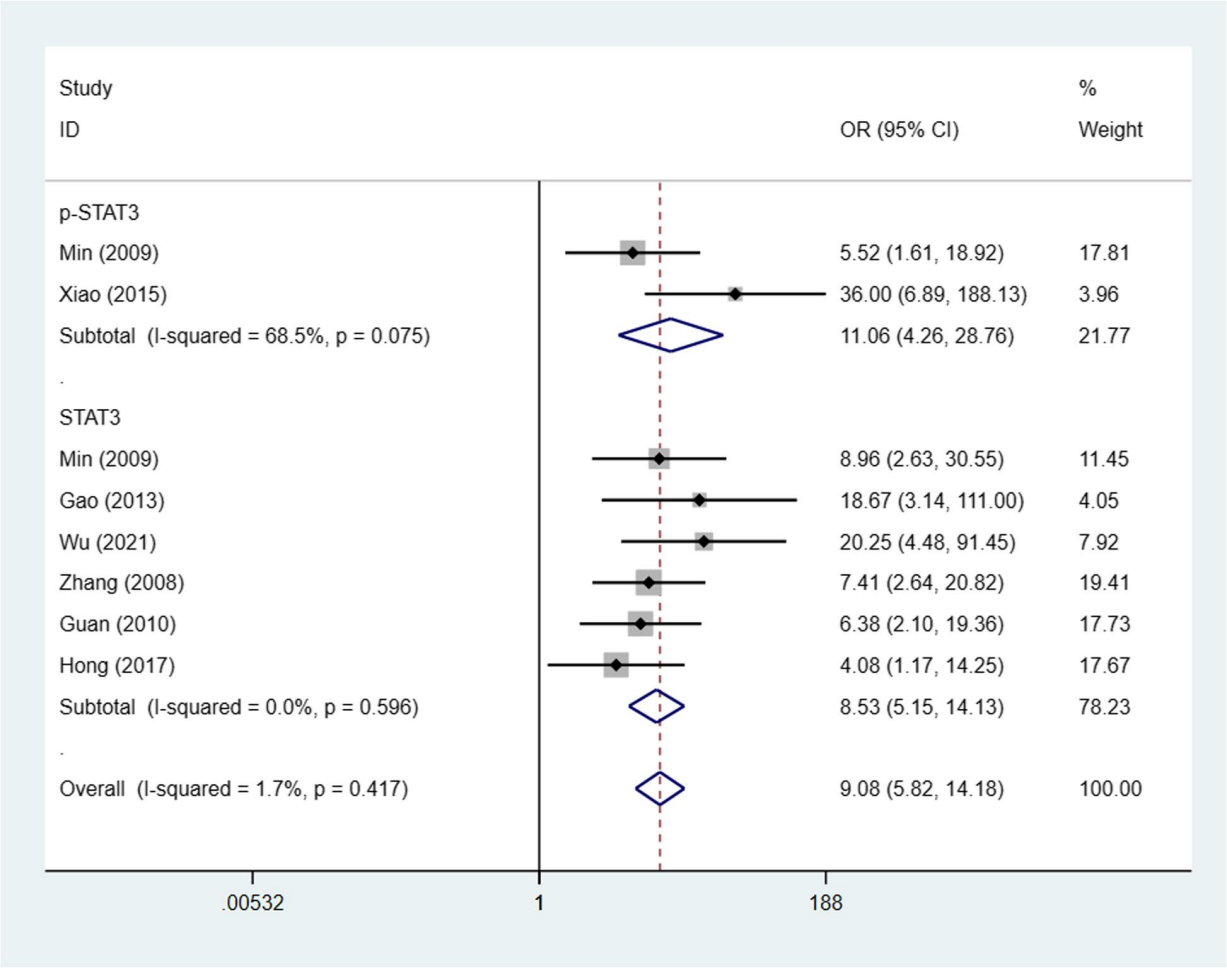
Forest plots of odds ratios for ovarian carcinoma vs. benign ovarian tumour. Abbreviation: OR, odds ratio

This meta-analysis comprised ovarian cancer and benign ovarian tumour STAT3/p-STAT3 expression data from 7 studies [22, 25–28, 34, 35] (8 trials). STAT3/p-STAT3 expression in ovarian carcinoma versus benign ovarian tumours was compared; the data were from 451 ovarian cancer cases and 164 patients suffering from benign ovarian tumours. A fixed effects model was adopted because there was no apparent interstudy heterogeneity (I^2^ = 1.7%, *p* = 0.417). These studies revealed that STAT3/p-STAT3 expression was significantly higher in ovarian carcinoma than in benign ovarian tumours (OR = 9.08, 95% CI = 5.82–14.18). We obtained consistent outcomes in the ovarian cancer biomarker subgroups (STAT3 and p-STAT3) (STAT3: OR = 8.53, 95% CI = 5.15–14.13, *p* < 0.00001; p-STAT3: OR = 11.06, 95% CI = 4.26–28.76, *p* < 0.00001) (Fig. 3). A sensitivity analysis was performed to evaluate the effect of individual studies on the pooled ORs by deleting single studies in turn. According to the sensitivity analysis, the identified significant difference was robust (Additional file 1: Fig. S2).

### STAT3/p-STAT3 expression in ovarian cancer versus borderline ovarian tumours

**Fig. 4:**
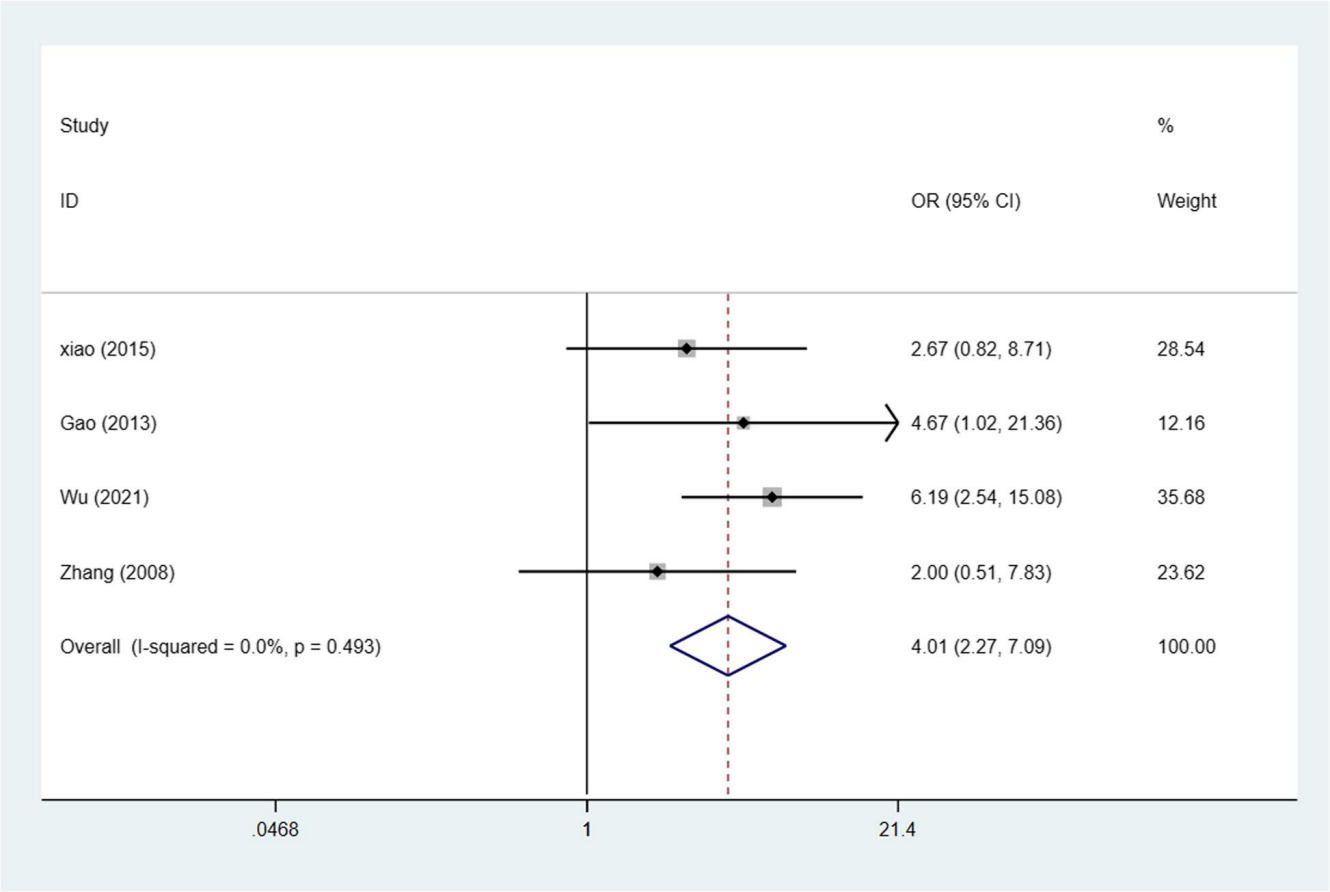
Forest plots of odds ratios for ovarian carcinoma vs. borderline ovarian tumours. Abbreviation: OR, odds ratio

This meta-analysis comprised ovarian cancer and borderline ovarian tumour STAT3/p-STAT3 expression data from 4 studies [25–28]. STAT3/p-STAT3 expression in ovarian carcinoma and borderline ovarian tumour was compared; the data were from 260 ovarian cancer cases and 78 borderline ovarian tumour cases. A fixed effects model was adopted because there was no apparent interstudy heterogeneity (*p* = 0.493, I^2^ = 0%). These studies revealed that STAT3/p-STAT3 expression was significantly higher in ovarian carcinoma than in borderline ovarian tumours (OR = 4.01, 95% CI = 2.27–7.09, *p* < 0.00001) (Fig. 4). A sensitivity was performed to evaluate the effect of individual studies on the pooled ORs by deleting single studies in turn. According to the sensitivity analysis, the identified significant difference was robust (Additional file 1: Fig. S3).

### STAT3/p-STAT3 expression and FIGO stage

**Fig. 5:**
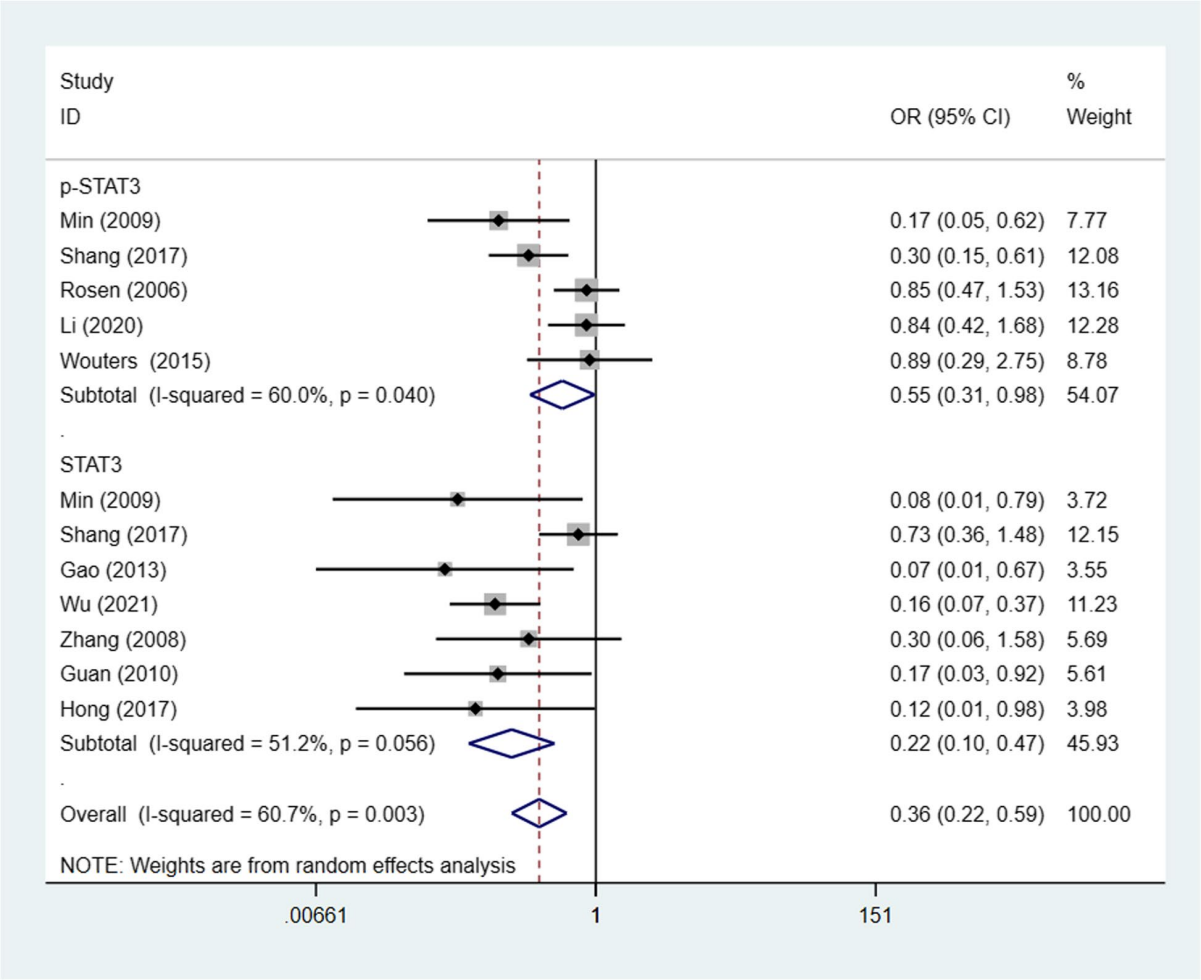
Forest plots of odds ratios for FIGO stage. Abbreviation: OR, odds ratio

Ten studies [22–24, 26–28, 30–32, 34, 35] (12 trials) investigated the association between STAT3/p-STAT3 expression level and FIGO stage (I-II vs. III/IV), with a combined total of 1243 patients. Because there was significant heterogeneity among the included studies (I^2^ = 60.7% *p* = 0.003), a random effects model was employed. The results demonstrated that compared with stage III-IV ovarian cancer patients, stage I–II ovarian cancer patients had significantly lower STAT3/p-STAT3 expression levels (OR = 0.36, 95% CI = 0.22–0.59, *p* < 0.00001). We obtained consistent outcomes for the ovarian cancer biomarker subgroups (STAT3 and p-STAT3) (STAT3: OR = 0.22, 95% CI = 0.10–0.47, p < 0.00001; p-STAT3: OR = 0.55, 95% CI = 0.31–0.98, *p* = 0.042) (Fig. 5). A sensitivity was performed to evaluate the effect of individual studies on the pooled ORs by deleting single studies in turn. According to the sensitivity analysis, this identified significant difference was robust (Additional file 1: Fig. S4). Owing to the identified heterogeneity, additional subgroup analyses in the meta-analysis were carried out. As summarized in Table 2, we proceeded with subgroup meta-analysis to identify the potential sources of heterogeneity by study region (Asia vs. non-Asia), year of publication (≥ 2010 vs < 2010), sample size (≥100 vs. < 100), scoring method (EI vs. non-EI) and the primary antibody used in IHC (rabbit antibody vs. others). The subgroup analysis findings were basically consistent with the overall results, and we did not find any sources of heterogeneity.

**Table 2:** Subgroup analysis of STAT3/p-STAT3 expression and FIGO stage of ovarian cancer

Stratification	Pooled OR (95% CI) random effects	I^**2**^ (%)	***p*** value of Q test
Year			
≤ 2010	0.487 (0.309–0.768)	60.2%	0.039
> 2010	0.408 (0.291–0.572)	63.6%	0.011
N pts			
< 100	0.147 (0.072–0.299)	0.0%	0.907
≥ 100	0.536 (0.397–0.722)	67.9%	0.008
Scoring method			
EI	0.417 (0.306–0.569)	58.4%	0.014
Non-EI	0.492 (0.282–0.859)	72.5%	0.026
Primary antibody			
Rabbit antibody	0.172 (0.032–0.925)	30.3%	0.208
Others	0.457 (0.323–0.645)	74.2%	0.002

### STAT3/p-STAT3 expression and tumour grade

**Fig. 6:**
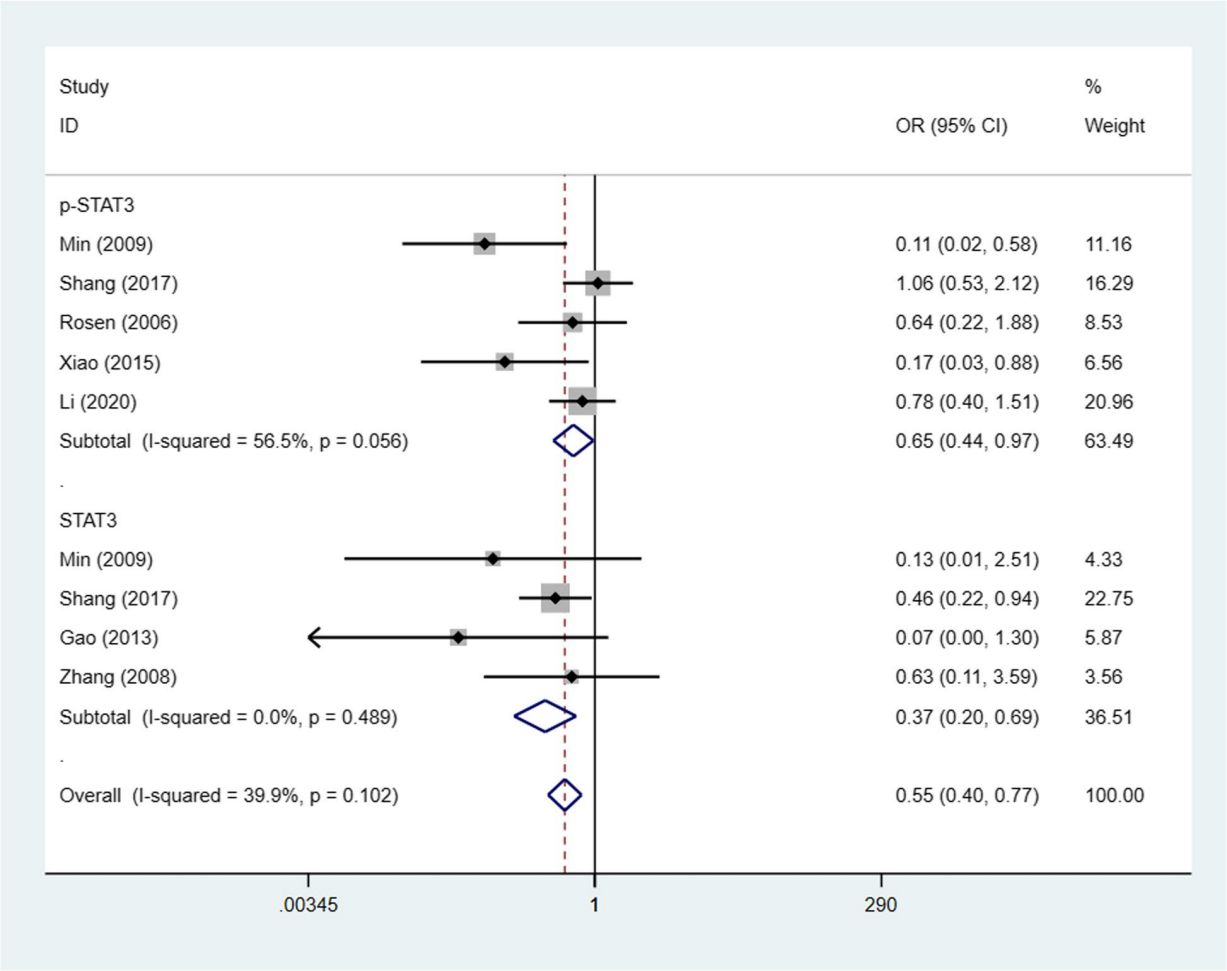
Forest plots of the odds ratios for tumour stage. Abbreviation: OR, odds ratio

Seven studies [22–26, 28, 30] (9 trials) investigated the association between STAT3/p-STAT3 expression level and tumour grade (G1 + G2 vs. G3), with a combined total of 953 patients. A fixed effects model was adopted because there was no apparent interstudy heterogeneity (I^2^ = 39.9%, *p* = 0.102). STAT3/p-STAT3 expression in G1 + G2 was significantly lower than that in G3 (OR = 0.55, 95% CI = 0.40–0.77, *p* < 0.00001). We obtained consistent outcomes in the ovarian cancer biomarker subgroups (STAT3 and p-STAT3) (STAT3: OR = 0.37, 95% CI = 0.20–0.69, *p* = 0.002; p-STAT3: OR = 0.65, 95% CI = 0.44–0.97, *p* = 0.035) (Fig. 6). A sensitivity analysis was performed to evaluate the effect of individual studies on the pooled ORs by deleting single studies in turn. According to the analysis, the identified significant difference was robust (Additional file 1: Fig. S5).

### STAT3/p-STAT3 expression and lymphatic metastasis

**Fig. 7:**
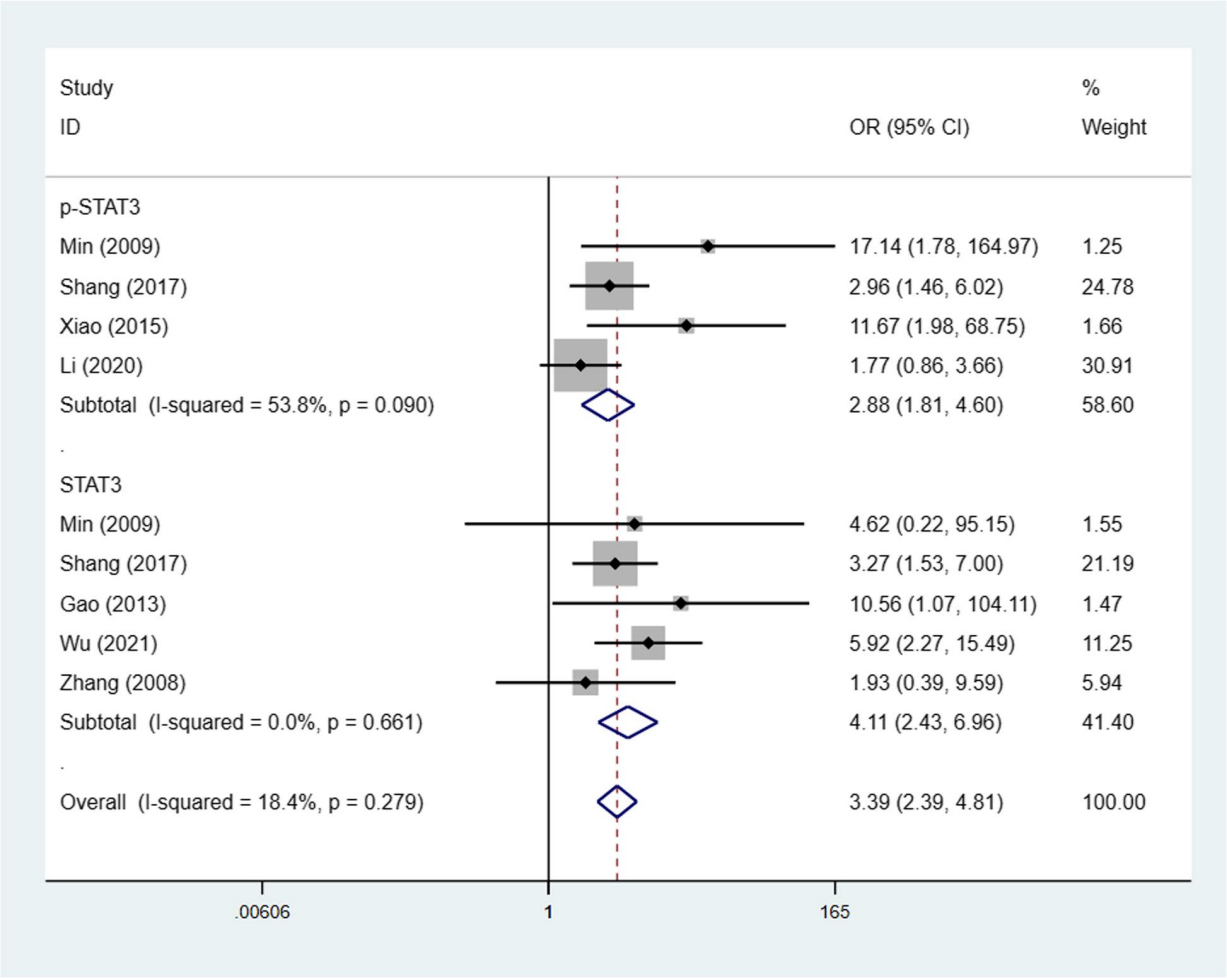
Forest plots of the odds ratios for lymphatic metastasis. Abbreviation: OR, odds ratio

Seven studies [22, 23, 25–28, 30] (9 trials) investigated the association between STAT3/p-STAT3 expression and lymph node metastasis (yes vs. no) among a combined 750 cases. A fixed effects model was adopted because there was no apparent interstudy heterogeneity (I^2^ = 18.4%, *p* = 0.279). The results showed significantly lower STAT3/p-STAT3 expression in lymphatic metastasis patients than in those without lymph node metastasis (OR = 3.39, 95% CI = 2.39–4.81, *p* < 0.00001). We obtained consistent outcomes in the ovarian cancer biomarker subgroups (STAT3 and p-STAT3) (STAT3: OR = 4.11, 95% CI = 2.43–6.96, *p* < 0.00001; p-STAT3: OR = 2.88, 95% CI = 1.81–4.60, *p* < 0.00001) (Fig. 7). A sensitivity was performed to evaluate the effect of individual studies on the pooled ORs by deleting single studies in turn. According to the sensitivity analysis, the identified significant difference was robust (Additional file 1: Fig. S6).

### STAT3/p-STAT3 expression and histological type

**Fig. 8:**
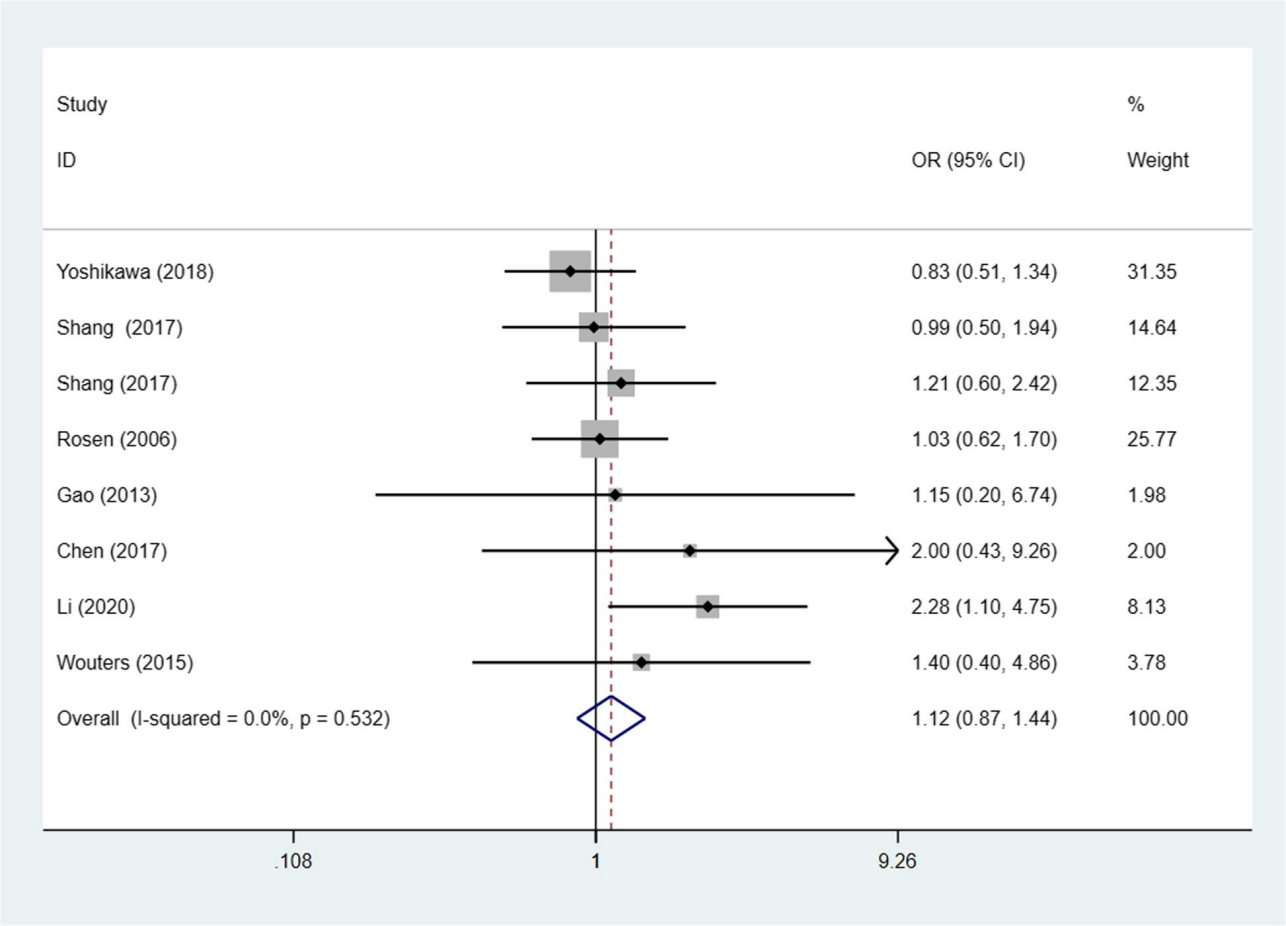
Forest plots of the odds ratios for histological type. Abbreviation: OR, odds ratio

**Fig. 9:**
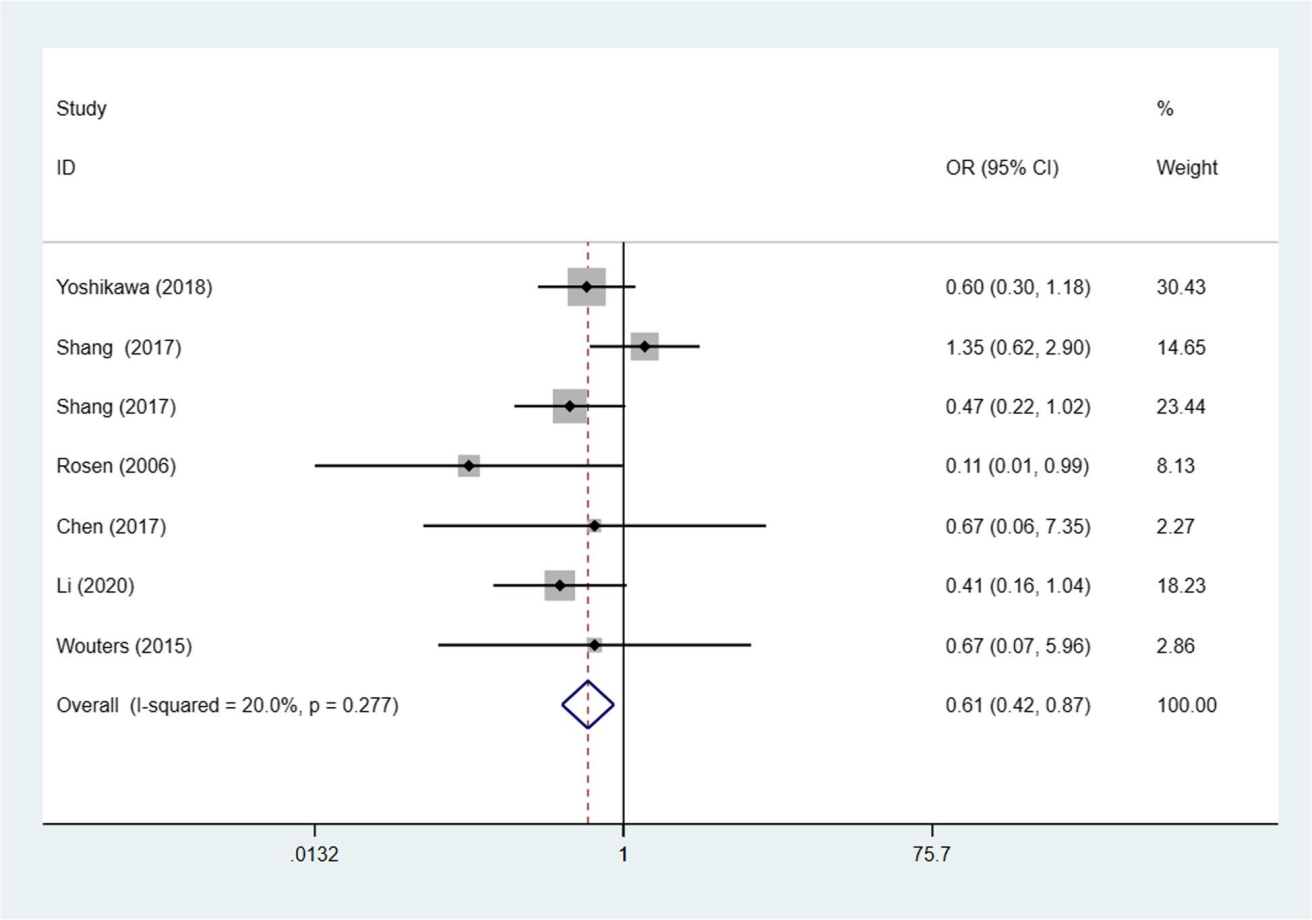
Forest plots of the odds ratios for histological type. Abbreviation: OR, odds ratio

**Fig. 10:**
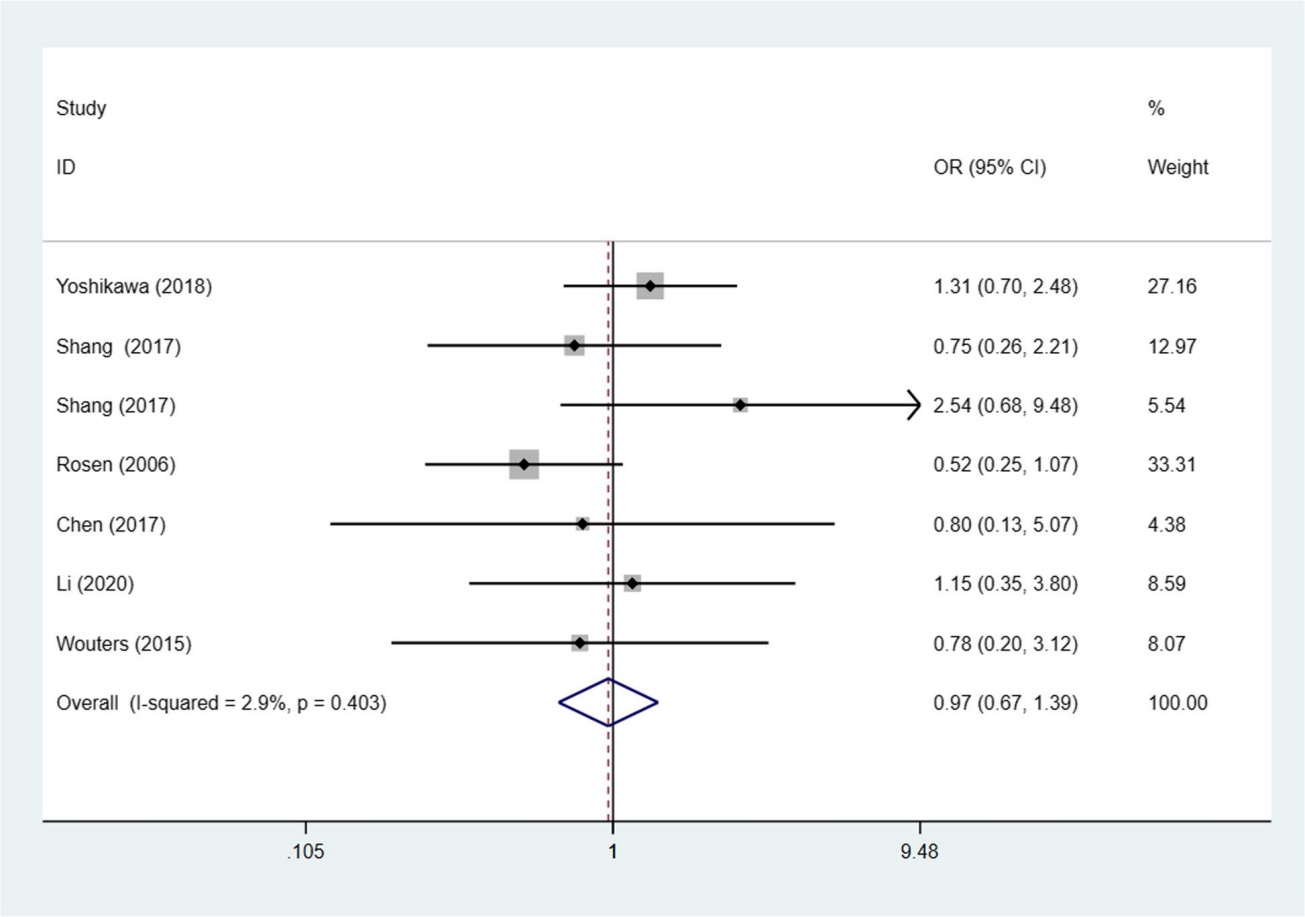
Forest plots of the odds ratios for histological type. Abbreviation: OR, odds ratio

**Fig. 11:**
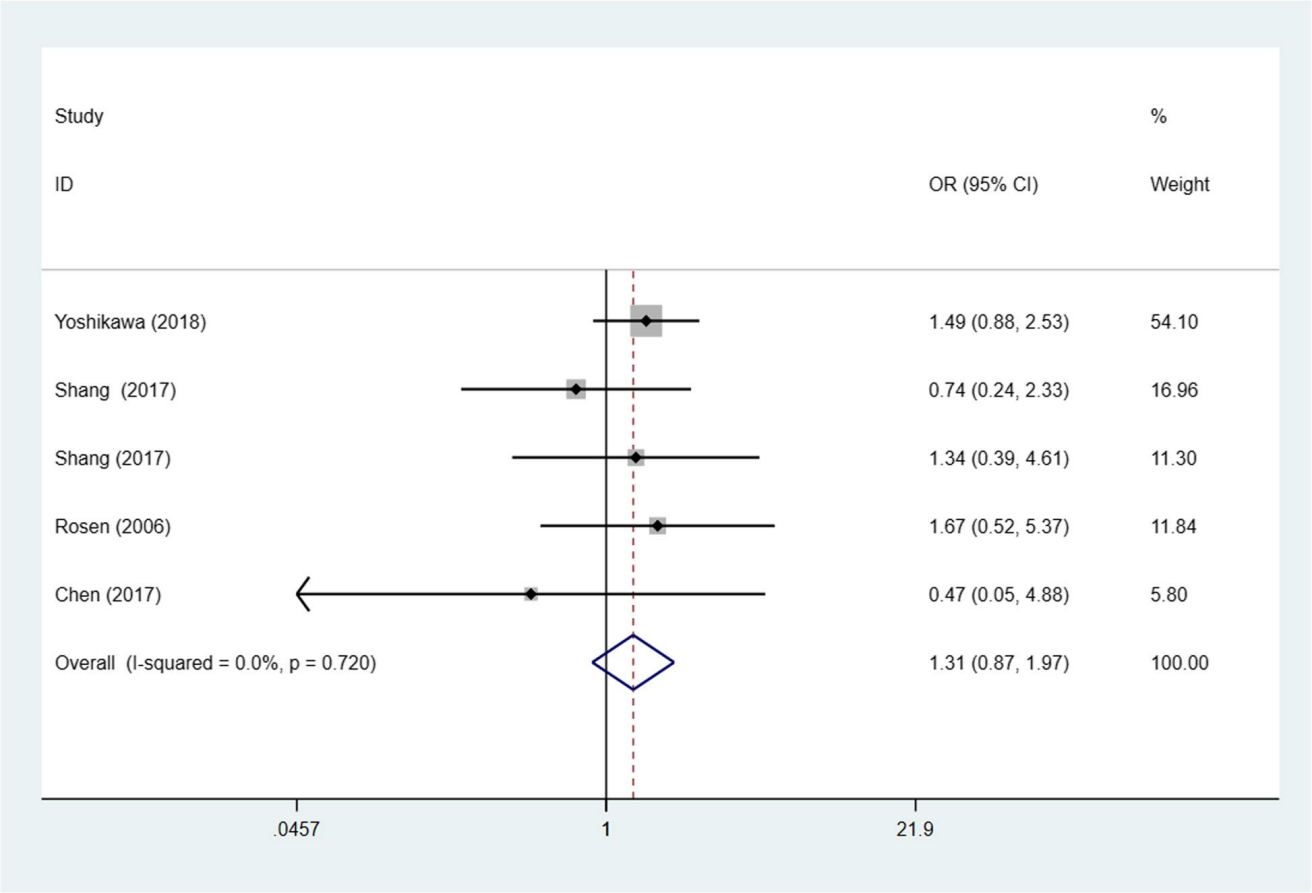
Forest plots of the odds ratios for histological type. Abbreviation: OR, odds ratio

Seven studies [20, 23, 24, 26, 29, 30, 32] (8 trials) investigated the association between STAT3/p-STAT3 expression levels and ovarian carcinoma subtype (serous vs. nonserous) among a combined 1238 patients. A fixed effects model was adopted because there was no apparent interstudy heterogeneity (*p* = 0.532, I^2^ = 0%). There was no significant difference in STAT3/p-STAT3 expression between serous and nonserous ovarian carcinoma patients (OR = 1.12, 95% CI =0.87–1.44, *p* = 0.374) (Fig. 8). Six studies [20, 23, 24, 29, 30, 32] (7 trials) investigated the association between STAT3/p-STAT3 expression and the subtype (mucinous vs. nonmucinous; endometrioid vs. nonendometrioid) of ovarian carcinoma in 1204 patients. A fixed effects model was applied because there was no obvious interstudy heterogeneity (mucinous vs. nonmucinous, *p* = 0.277, I^2^ = 20.0%) (endometrioid vs. nonendometrioid *p* = 0.403, I^2^ = 2.9%). STAT3/p-STAT3 expression was significantly lower in patients with mucinous ovarian cancer than in patients with nonmucinous ovarian cancer (OR = 0.61, 95% CI = 0.42–0.87, *p* = 0.007) (Fig. 9). There was no significant difference in STAT3/p-STAT3 expression between endometrioid and nonendometrioid ovarian carcinoma patients (OR = 0.97, 95% CI = 0.67–1.39, *p* = 0.853) (Fig. 10). Four studies [20, 23, 24, 29] (5 trials) investigated the association between STAT3/p-STAT3 expression and ovarian carcinoma subtype (clear cell vs. non-clear cell) in 966 patients. A fixed effects model was used because there was no obvious interstudy heterogeneity (*p* = 0.720, I^2^ = 0%). There was no significant difference in STAT3/p-STAT3 expression between clear cell and non-clear cell ovarian carcinoma patients (OR = 1.31, 95% CI =0.87–1.97, *p* = 0.196) (Fig. 11). A sensitivity analysis was performed to evaluate the effect of each study on the pooled ORs by removing each study in turn. According to the analysis, the lack of a significant difference was robust (Additional file 1: Fig. S7).

### STAT3/p-STAT3 expression and OS

**Fig. 12:**
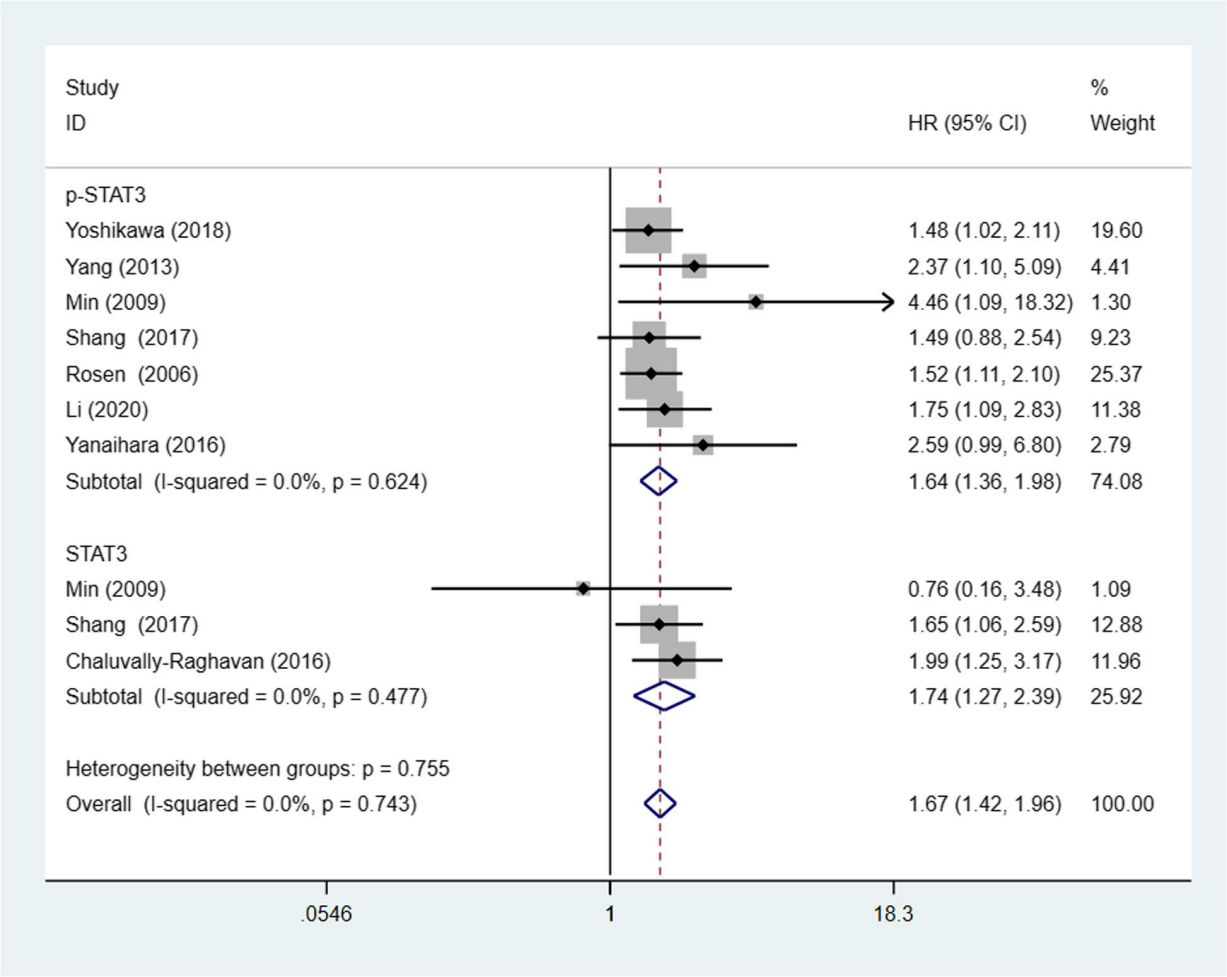
Forest plot of hazard ratios for overall survival. Abbreviation: HR, hazard ratio

Eight studies [20–24, 30, 31, 33] (10 trials) with a combined total of 1450 patients explored the relevance of STAT3/p-STAT3 expression to OS. A fixed effects model was used because there was no obvious interstudy heterogeneity (*p* = 0.743, I^2^ = 0%). STAT3/p-STAT3 expression was significantly associated with worse overall survival in patients (HR = 1.67, 95% CI: 1.42–1.96, *p*<0.00001). Consistent outcomes were found in the ovarian cancer biomarker subgroups (STAT3 and p-STAT3) (STAT3: HR =1.74, 95% CI =1.27–2.39, *p* = 0.001; p-STAT3: HR = 1.64, 95% CI = 1.36–1.98, *p* < 0.0001) (Fig. 12). A sensitivity analysis was performed to evaluate the effect of individual studies on the pooled HRs by deleting each study in turn. According to the analysis, this significant difference was robust (Additional file 1: Fig. S8).

### STAT3/p-STAT3 expression and PFS

**Fig. 13:**
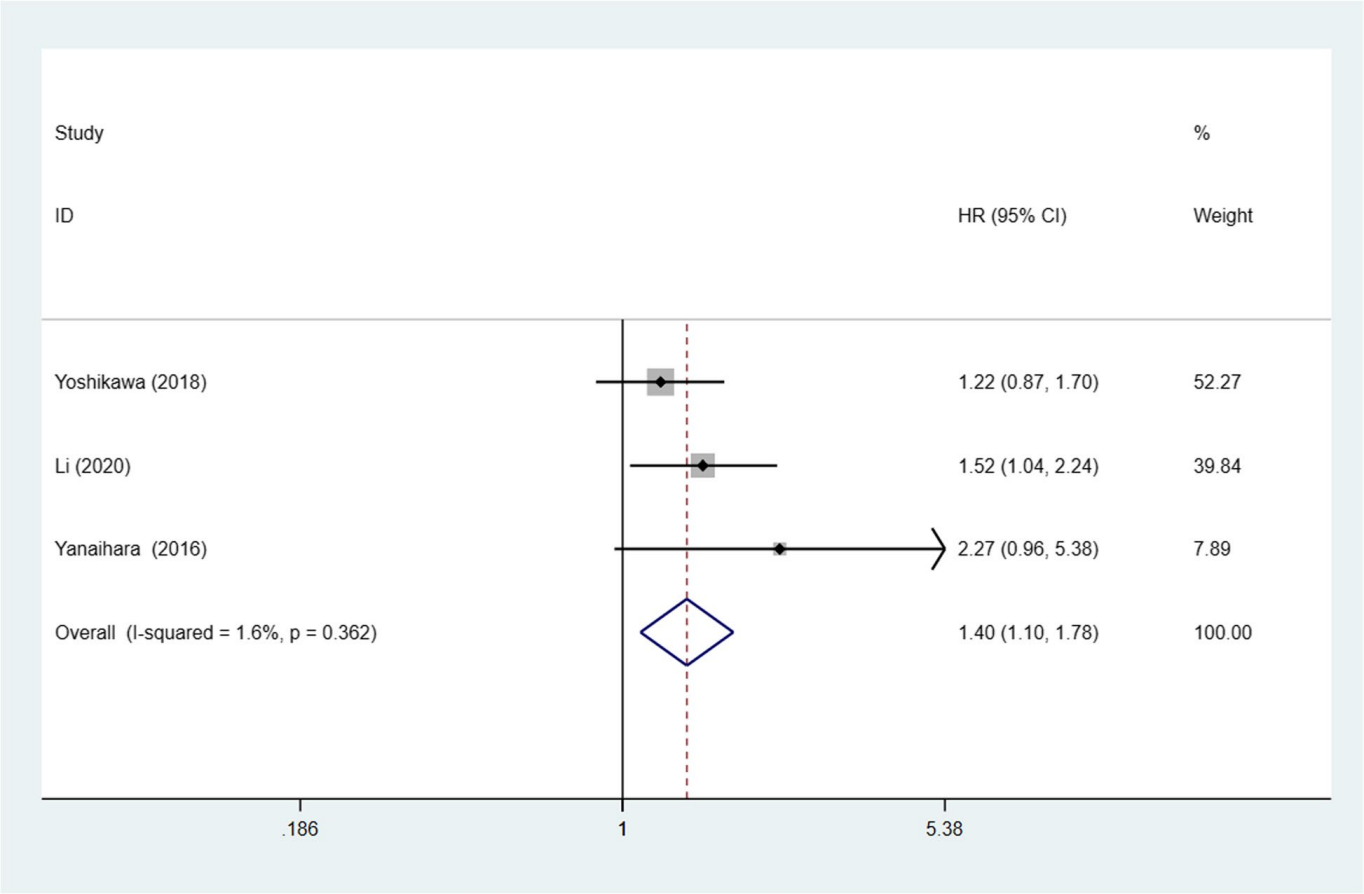
Forest plot of hazard ratios for progression-free survival. Abbreviation: HR, hazard ratio

Three studies [20, 30, 31] with a combined total of 581 patients investigated the relationship between STAT3/p-STAT3 expression and PFS. A fixed effects model was used because there was no obvious interstudy heterogeneity (*p* = 0.362, I^2^ = 1.6%). STAT3/p-STAT3 expression was found to be significantly associated with worse PFS (HR = 1.40, 95% CI: 1.10–1.78, *p* = 0.007) (Fig. 13). A sensitivity analysis was performed to evaluate the effect of individual studies on the pooled HRs by deleting each study in turn. According to the analysis, this significant difference was robust (Additional file 1: Fig. S9).

**Fig. 14:**
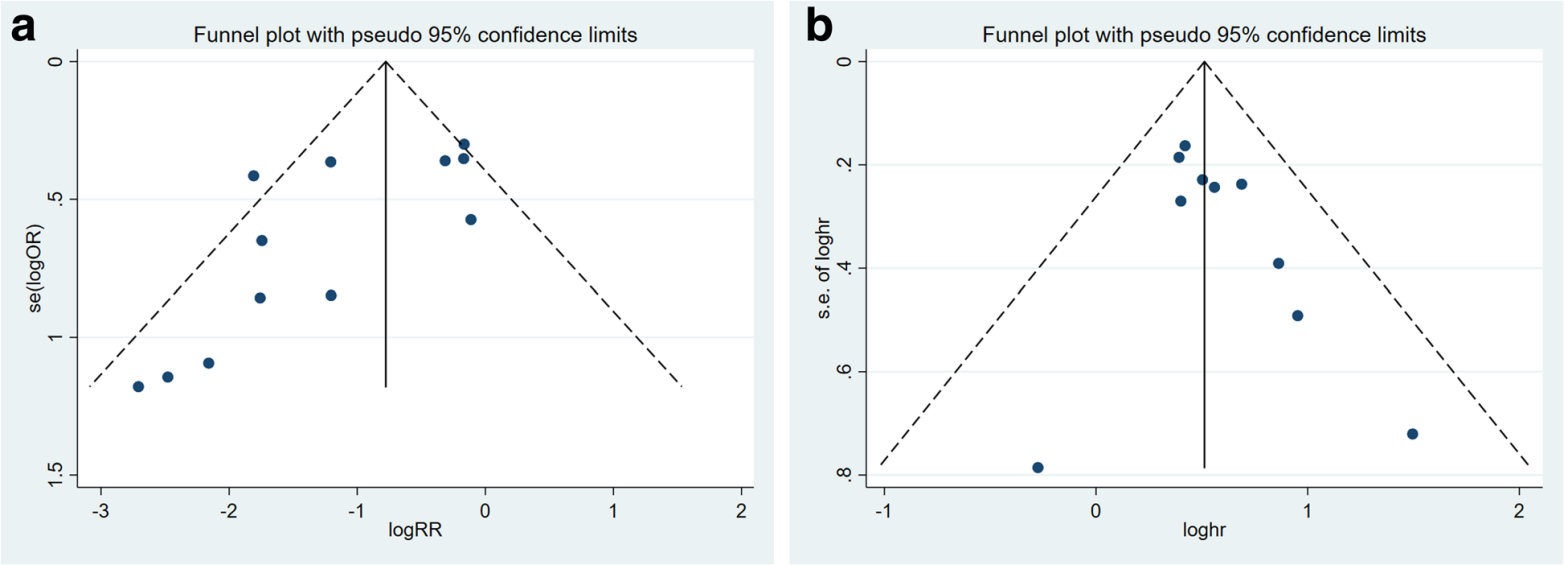
Funnel plots for publication bias regarding FIGO stage (**A**) and overall survival (**B**)

We constructed funnel plots and carried out the Begg test and Egger’s test for outcomes from ≥10 studies. In evaluating the relevance of p-STAT3/STAT3 expression on FIGO stage, the funnel plot revealed some dissymmetry, and Egger’s test with the Begg test suggested potential publication bias (Egger: *p* = 0.024, Begg: *p* = 0.016). In evaluating the relevance of p-STAT3/STAT3 expression in OS, the funnel plot revealed no proof of asymmetry, and Egger’s test with the Begg test suggested no potential publication bias (Egger: *p* = 0.189, Begg: *p* = 0.210) (Fig. 14).

## Discussion

In the current meta-analysis, we assessed the influence of STAT3/p-STAT3 expression on the clinicopathological features and prognosis of ovarian cancer patients, and 16 published articles were included. The outcomes demonstrated that high STAT3/p-STAT3 expression in ovarian cancer tissue was correlated with a higher FIGO stage, a more advanced tumour grade, a higher risk of lymph node metastasis, non-mucinous ovarian cancer, shorter overall survival, and shorter progression-free survival. Min, Xiao, Gao, and Wu, [22, 25–27] found that compared with normal ovarian tissue and borderline and benign tumours, the expression level of STAT3/p-STAT3 in ovarian cancer was significantly higher. The meta-analysis results suggested that ovarian cancer tissue had higher STAT3/p-STAT3 expression than that of normal tissue and borderline and benign tumours. This result revealed that the occurrence of ovarian cancer was positively related to STAT3/p-STAT3 expression. This may explain why the constitutive activation of STAT3/p-STAT3 is common in tumour cells but is not often seen in normal cells.

In this study, we investigated the relationship between STAT3/p-STAT3 expression and 3 major pathological characteristics (FIGO stage, tumour grade, and lymph node metastasis). Rosen [24] found no relationship between the p-STAT3 expression level and the FIGO stage or tumour grade. Li [30] found no relationship between the p-STAT3 expression level and the FIGO stage, tumour grade or lymph node metastasis. Shang [23] found that the p-STAT3 expression level was associated with FIGO stage and lymph node metastasis and that STAT3 expression correlated with tumour grade and lymph node metastasis**.** Persistent STAT3 activation promotes tumour progression and metastasis in various cancers [36–40]. Our study demonstrated that higher STAT3 and p-STAT3 expression was correlated with FIGO stage, tumour grade, and lymph node metastasis. These findings indicate that the STAT3 and p-STAT3 expression levels could be independent prognostic factors for ovarian cancer.

The histologic subtypes of ovarian cancer are classified into serous, mucinous, endometrioid, and clear-cell carcinoma. Rosen [24] found an association between p-STAT3 expression and poorly differentiated (75%), clear cell (73%), and serous carcinoma (63%) histotypes (*p* = 0.01) but not with any of the other clinicopathologic variables tested. In the present study, we found that STAT3/p-STAT3 expression was significantly different in patients with serous vs. non-serous, endometrioid vs. non-endometrioid, and clear cell vs. non-clear cell ovarian cancer subtypes. There was no significant difference in STAT3/p-STAT3 expression between mucinous vs. non-mucinous ovarian cancer subtypes.

In addition, we comprehensively analysed the relationship of STAT3/p-STAT3 expression with OS and PFS. High STAT3/p-STAT3 expression was significantly associated with poor OS and unfavourable PFS in ovarian cancer patients. Cui [41] reported that higher STAT3 expression was correlated with shorter OS. However, Wu [42] found no significant correlation between STAT3 expression and ovarian cancer. Such disagreement may result from differences in the included literature.

To the best of our knowledge, this is the first meta-analysis to systematically explore the relevance of STAT3/p-STAT3 expression on the prognosis and clinicopathological characteristics of ovarian cancer. The included 16 studies covering 1747 ovarian cancer patients led to more reliable and stable results compared with those of the individual studies. However, our study also has some limitations. First, most populations included in our analysis were Asian, with only 4 studies carried out with Caucasian patients. Therefore, our results should be confirmed with additional research in other ethnicities. Second, our research strategy was restricted to articles from four databases (PubMed, Embase, CNKI, and Wan Fang) and English and Chinese publications only. Therefore, selection bias in the outcomes cannot be entirely excluded. Third, different studies used different scoring methods to define high STAT3/p-STAT3 expression. Some studies relied on both the intensity of staining and the percentage of stained cells to evaluate STAT3/p-STAT3 expression, while others relied on the percentage of positive cells alone. Fourth, different antibodies, dilutions, and cut-off values may have resulted in heterogeneity and affected the study results. Therefore, uniform criteria must be applied when determining STAT3/p-STAT3 expression to more reliably interpret its significance in ovarian cancer. Fifth, due to the obvious heterogeneity in the FIGO stage analysis, we conducted subgroup analysis based on year of publication, scoring method, sample size, and primary antibody to identify the source of heterogeneity. However, the heterogeneity remained and its source could not be identified. Sixth, the funnel plot, Egger’s test, and the Begg test indicated publication bias in the FIGO stage, leading to overestimating the effect sizes. Finally, several original studies did not report HRs with their 95% CIs or the estimated HRs and their 95% CIs from Kaplan–Meier survival curves, which may have introduced bias.

Here, we summarized all relevant studies and performed a meta-analysis to assess the value of STAT3/p-STAT3 expression as a prognostic indicator for OC patients. Despite the aforementioned limitations, the results of this meta-analysis indicated the prognostic value and clinicopathological significance of STAT3 expression in ovarian cancer. Our findings show that STAT3 expression has potential as a specific biomarker in patients with ovarian cancer, and its increased expression indicates poor patient prognosis. These results may contribute to future explorations of the pathogenesis, diagnosis, anti-STAT3 therapy, and prognosis in ovarian cancer.

## Supplementary Information

**Additional file 1: Figure S1.** Sensitivity analysis for ovarian carcinoma vs. normal ovarian tissue. **Figure S2.** Sensitivity analysis for ovarian carcinoma vs. benign ovarian tumour. **Figure S3.** Sensitivity analysis for ovarian carcinoma vs. borderline ovarian tumours. **Figure S4.** Sensitivity analysis for FIGO stage. **Figure S5.** Sensitivity analysis for tumour stage. **Figure S6.** Sensitivity analysis for lymphatic metastasis. **Figure S7.** Sensitivity analysis for histological type (serous vs. non-serous) (A). Sensitivity analysis for histological type (mucinous vs. non-mucinous) (B). Sensitivity analysis for histological type (endometrioid vs. non-endometrioid) (C). Sensitivity analysis for histological type (clear cell vs non- clear cell)(D). **Figure S8.** Sensitivity analysis for overall survival. **Figure S9.** Sensitivity analysis for progression‐free survival.

## Data Availability

Not applicable.

## Abbreviations

FIGO: International Federation of Gynecology and Obstetrics
OS: Overall survival
STAT: Signal transducers and activators of transcription
STAT3: Signal transducer and activator of transcription 3
JAK: Janus kinase
IHC: Immunohistochemical
HR: Hazard ratio
CI: Confidence interval
NOS: Newcastle–Ottawa Quality Assessment Scale
OR: Odds ratio
PFS: Progression-free survival
