# Supplementary material for: The impact of STAT3 and phospho-STAT3 expression on the prognosis and clinicopathology of ovarian cancer: a systematic review and meta-analysis
Source: J Ovarian Res. 2021 Nov 18;14:164. doi: 10.1186/s13048-021-00918-6 (PMC8600722; doi:10.1186/s13048-021-00918-6)
Supplement: Supplementary file 1 — Additional file 1: Figure S1. Sensitivity analysis for ovarian carcinoma vs. normal ovarian tissue. Figure S2. Sensitivity analysis for ovarian carcinoma vs. benign ovarian tumour. Figure S3. Sensitivity analysis for ovarian carcinoma vs. borderline ovarian tumours. Figure S4. Sensitivity analysis for FIGO stage. Figure S5. Sensitivity analysis for tumour stage. Figure S6. Sensitivity analysis for lymphatic metastasis. Figure S7. Sensitivity analysis for histological type (serous vs. non-serous) (A). Sensitivity analysis for histological type (mucinous vs. non-mucinous) (B). Sensitivity analysis for histological type (endometrioid vs. non-endometrioid) (C). Sensitivity analysis for histological type (clear cell vs non- clear cell)(D). Figure S8. Sensitivity analysis for overall survival. Figure S9. Sensitivity analysis for progression‐free survival. [file 13048_2021_918_MOESM1_ESM.zip › Additional file1.doc]

**Additional file 1: Figure S1 Sensitivity analysis for ovarian carcinoma vs. normal ovarian tissue.**

**Additional file 1: Figure S2 Sensitivity analysis for ovarian carcinoma vs. benign ovarian tumour.**

**Additional file 1: Figure S3 Sensitivity analysis for ovarian carcinoma vs. borderline ovarian tumours.**

**Additional file 1: Figure S4 Sensitivity analysis for FIGO stage.**

**Additional file 1: Figure S5 Sensitivity analysis for tumour stage.**

**Additional file 1: Figure S6 Sensitivity analysis for lymphatic metastasis.**

**Additional file 1: Figure S7 Sensitivity analysis for histological type (serous vs. non-serous) (A).**

**Additional file 1: Figure S7 Sensitivity analysis for histological type (mucinous vs. non-mucinous) (B).**

**Additional file 1: Figure S7 Sensitivity analysis for histological type (endometrioid vs. non-endometrioid) (C).**

**Additional file 1: Figure S7 Sensitivity analysis for histological type (clear cell vs non- clear cell）(D).**

**Additional file 1: Figure S8 Sensitivity analysis for overall survival.**

**Additional file: Figure S9 Sensitivity analysis for progression‐free survival.**
